# From Macro to Micro: Comparison of Imaging Techniques to Detect Vascular Network Formation in Left Ventricle Decellularized Extracellular Matrix Hydrogels

**DOI:** 10.3390/gels8110729

**Published:** 2022-11-10

**Authors:** Meng Zhang, Vasilena E. Getova, Francisco Drusso Martinez-Garcia, Theo Borghuis, Janette K. Burgess, Martin C. Harmsen

**Affiliations:** 1 University of Groningen, University Medical Center Groningen, Department of Pathology and Medical Biology, Hanzeplein 1 (EA11), 9713 GZ Groningen, The Netherlands; 2 University of Groningen, University Medical Center Groningen, W.J. Kolff Research Institute, A. Deusinglaan 1, 9713 AV Groningen, The Netherlands; 3 University of Groningen, University Medical Center Groningen, Groningen Research Institute for Asthma and COPD (GRIAC), Hanzeplein 1 (EA11), 9713 AV Groningen, The Netherlands

**Keywords:** vascular network formation, fluorescence microscopy, confocal microscopy, optical coherence tomography, decellularized extracellular matrix hydrogels

## Abstract

**Background:** Angiogenesis is a crucial process in physiological maintenance and tissue regeneration. To understand the contribution of angiogenesis, it is essential to replicate this process in an environment that reproduces the biochemical and physical properties which are largely governed by the extracellular matrix (ECM). We investigated vascularization in cardiac left ventricular ECM hydrogels to mimic post-myocardial repair. We set out to assess and compare different destructive and non-destructive methods, optical as well as non-optical, to visualize angiogenesis and associated matrix remodeling in myocardial ECM hydrogels. **Methods:** A total of 100,000, 300,000, and 600,000 Human Pulmonary Microvascular Endothelial Cells (HPMEC) were seeded in left ventricular cardiac ECM hydrogel in 48-well plates. After 1, 7, and 14 days of culture, the HPMEC were imaged by inverted fluorescence microscopy and 3D confocal laser scanning microscopy (Zeiss Cell Discoverer 7). In addition, cell-seeded ECM hydrogels were scanned by optical coherence tomography (OCT). Fixed and paraffin-embedded gels were thin-sectioned and assessed for ECM components via H&E, picrosirius red histochemical staining, and immunostaining for collagen type I. ImageJ-based densitometry was used to quantify vascular-like networks and GraphPad was used for statistical analyses. **Results:** Qualitative analyses were realized through fluoromicrographs obtained by the confocal laser scanning microscope which allowed us to visualize the extensive vascular-like networks that readily appeared at all seeding densities. Quantification of networks was only possible using fluoromicrographs from inverted microscopy. These showed that, after three days, the number of master junctions was seeding density-dependent. The resolution of optical coherence tomography was too low to distinguish between signals caused by the ECM and cells or networks, yet it did show that gels, irrespective of cells, were heterogeneous. Interestingly, (immuno)histochemistry could clearly distinguish between the cast cardiac-derived matrix and newly deposited ECM in the hydrogels. The H&E staining corroborated the presence of vascular-like network structures, albeit that sectioning inevitably led to the loss of 3D structure. **Conclusions:** Except for OCT, all methods had complementary merit and generated qualitative and quantitative data that allowed us to understand vascular network formation in organ-derived ECM hydrogels.

## 1. Introduction

Cell culture is an important approach to modeling physiological processes in vitro. Traditionally, cell culture is performed as two-dimensional monolayers on flat and very stiff tissue culture-treated polystyrene surfaces (TCPS). In 2D culturing, propagation, manipulation, and harvest of cells poses no challenge. In fact, the high stiffness drives proliferation of cells which renders them in a rather unphysiological state. Yet, TCPS suits the replication of hard tissues in our body, such as bone and cartilage [1]. To some extent, it facilitates the polarization of cells such as epithelial and endothelial cells [2]. However, soft tissue, as well as bone and cartilage, is organized in a three-dimensional fashion, in which cells are surrounded by an extracellular matrix (ECM) [3]. Moreover, the stiffness of ECM in tissues and organs such as the heart is orders of magnitude lower than TCPS, which adds to physiological behavior.

Mimicking the cellular organization and function in tissues and organs demands a three-dimensional culture system. In a simplified view, tissues comprise cells embedded in the ECM. The native ECM is composed of a mixture of mostly fibrillar proteins embedded in a gel of highly negatively charged water-retaining polysaccharides. With advanced techniques involving detergents and treatment with high salt solutions, tissues are readily decellularized while retaining the (decellularized) ECM that is akin to the normal tissue ECM [4,5,6]. Proteomic analysis on the cardiac ECM hydrogel has resulted in a more complete picture of the molecular composition, which includes various collagens, laminins, versican, and more [6]. Native cardiac ECM is a promising biomedical material that can be applied to reconstructive surgery, due to its structural and functional resemblance to native tissue [7]. Additionally, native cardiac ECM has been used as a scaffold for 3D culturing [8,9]. Previous research has shown that injectable biohybrid ECM-based hydrogels promote cardiac tissue regeneration [10].

Angiogenesis is a crucial process in physiological tissue maintenance and repair and includes migration, proliferation, differentiation, and maturation of endothelial cells, as well as remodeling of the ECM by these cells. In pathophysiology, angiogenesis contributes to fibrosis, tumorigenesis [11], and chronic inflammation [12]. Angiogenesis depends on and is regulated by endothelial cell interaction with the surrounding three-dimensional ECM environment [13]. Endothelial cells sprout from a root vessel, migrate, proliferate, then align and ultimately form tubes [14] under certain stimuli. Natural ECM is a reservoir of various growth factors that drive angiogenesis, such as the vascular endothelial growth factors (VEGFs) family and the platelet-derived growth factor (PDGF) family [15,16]. Less well studied but no less relevant, endothelial cells sense and respond to the continuous changes in mechanical forces via ECM in the microenvironment [17]. To understand the contribution of angiogenesis to physiological repair and regeneration, and evaluate the effect of anti- and pro-angiogenesis, it is essential to replicate this process in a specific organ-derived ECM environment. Previous research has shown that kidney ECM enhanced the vascularization of the human kidney [18]. There are no published studies on the angiogenesis of only endothelial cells in cardiac left ventricular (LV) ECM hydrogels. Our previous study showed that confluent monolayers of primary adipose tissue-derived mesenchymal stromal cells (ASC) support vascular network formation (VNF) by human umbilical vein endothelial cells (HUVEC) in 3D upon seeding in Matrigel [7]. Furthermore, we proved that LV ECM hydrogel supports co-culture and vascular network formation of ASC with human pulmonary microvascular endothelial cells (HPMEC) [6].

Several distinct technologies allow for imaging and assessment of cell behavior in 3D ECM-cultured cells, each with benefits and limitations. Inverted light/fluorescence microscopy is most commonly used to image 2D-cultured cells to monitor cellular processes such as migration or endothelial sprouting in real-time [19]. A limitation is the shallow depth of field (DoF) which limits the maximum focusing depth in 3D cultures. Immunohistochemical staining of thin tissue sections, often around 4 µm, of fixed and paraffin-embedded 3D tissues or hydrogel cultures allows for the acquisition of a single layer of the vascular network formed in one plane. To generate a 3D reconstruction of the network requires the assembly of a large number of serial (optical) sections, which is challenging. Optical coherence tomography (OCT) is a noninvasive real-time 3D optical imaging technology based on light refraction. It is commonly used clinically to assess airways or the placement of stents in arteries. In principle, OCT has a submillimeter to micrometer resolution, which might suffice to visualize networks. Additionally, OCT allows repetitive measurements of 3D cultures over time so as to follow the formation of networks [20]. Thus, fluorescence inverted microscopy, stacked confocal laser scanning microscopy, and OCT allow for repeated measurements, while destructive methods that rely on embedding, sectioning, and staining do not. However, we expect that stained sections harbor the highest optical resolution, albeit no more than a ‘2D’ plane at a time. A distinct advantage of staining thin sections would be that matrix remodeling can be readily assessed by, for example, picrosirius red staining.

In the present study, we investigated the spatiotemporal dynamics of vascular network formation (VNF) by microvascular cells and HPMEC in cardiac left ventricle (LV) ECM. We set out to assess the suitability of imaging methods for visualizing VNF by HPMEC during 3D culture as a prelude to future research.

## 2. Results and Discussion

### 2.1. Results

#### 2.1.1. Vascular Structure Is Shown by Inverted Fluorescent Microscopy

**Figure 1:**
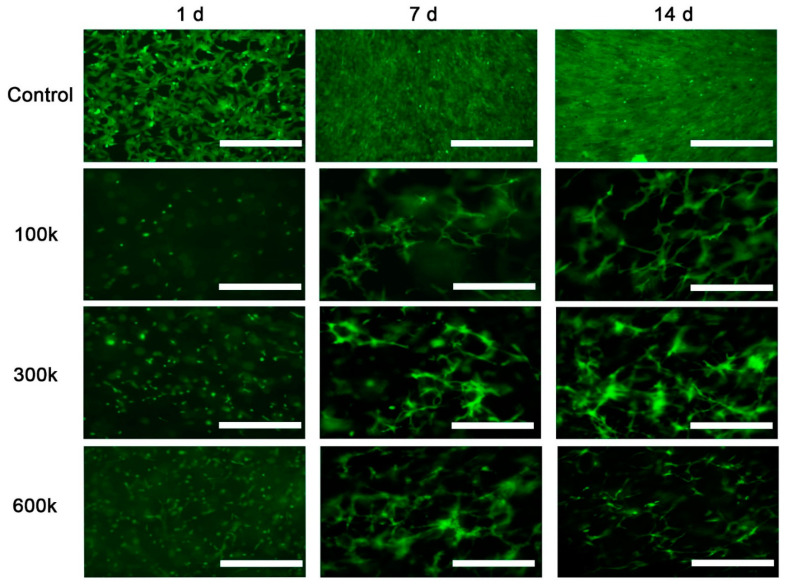
Vascular network formation (VNF) by HPMEC detected by fluorescent microscopy imaging; 100 k, 300 k, and 600 k HPMEC in LV ECM hydrogel were examined after 1, 7, and 14 days. As control, HPMEC were seeded directly on culture plastic. Scale bars represent 400 µm.

**Figure 2:**
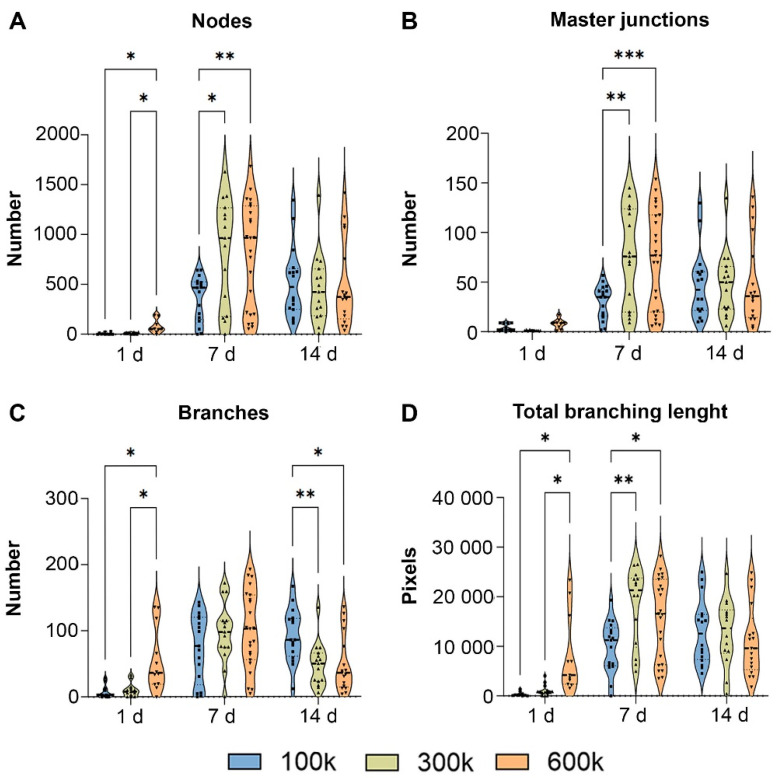
Quantitative analyses of VNF by HPMEC in LV ECM hydrogels. (**A**): comparison of the number of nodes based on ImageJ quantification among 100 k, 300 k, and 600 k HPMEC at three time points (1 d, 7 d, and 14 d). (**B**): comparison of the number of master junctions based on ImageJ quantification among 100 k, 300 k, and 600 k HPMEC at three time points (1 d, 7 d, and 14 d). (**C**): comparison of the number of branches based on ImageJ quantification among 100 k, 300 k, and 600 k HPMEC at three time points (1 d, 7 d, and 14 d). (**D**): comparison of total branching length based on ImageJ quantification among 100 k, 300 k, and 600 k HPMEC at three time points (1 d, 7 d, and 14 d). * *p* < 0.05, ** *p* < 0.01, **** *p* <<0.0001.

Inverted fluorescence microscopy imaging showed that VNF of HPMEC occurred spontaneously in the ECM hydrogel (Figure 1). In contrast, cells seeded on the surface of tissue culture plastic kept proliferating and became confluent on day 7 (Figure 1, upper row). In fact, HPMEC overgrew each other at day 14 and never engaged in VNF. Unlike HPMEC seeded on TCPS that had a spindle-shaped appearance, HPMEC inside hydrogels remained rounded on day 1, except for 600 k HPMEC where few spindles were observed (Figure 1, left column). At all three seeding densities, VNF by HPMEC had occurred in hydrogels by day 7 (Figure 1, middle column) and appeared to have expanded on day 14 (Figure 1, right column). The fluoromicrographs showed networks at different depths, yet were visualized as a ‘flattened’ image, i.e., in a single plane captured by the microscope’s camera. Therefore, ImageJ could not discriminate between ‘genuine’ junctions and tubes that were positioned perpendicular to each other at different planes. Because this is a systemic error, i.e., occurring in all samples, we continued our analyses. VNF increased in a seeding density-dependent manner (Figure 2): on day 7, 100 k HPMEC had produced 363 ± 55 nodes, which was less than the 830 ± 139 nodes in 300 k HPMEC (*p* < 0.05) and 805 ± 110 nodes in 600 k HPMEC (*p* < 0.01) (Figure 2A). Additionally, 100 k HPMEC had produced 31 ± 4 master junctions, which was less than the 77 ± 13 junctions in 300 k HPMEC (*p* < 0.01) and also less than the 73 ± 11 junctions in 600 k HPMEC (*p* < 0.001) (Figure 2B). It showed that there was no difference between the three groups for the number of branches on day 7; however, on day 14, the number of branches for 100 k HPMEC was 93 ± 10, which was higher than the 48 ± 8 observed in 300 k HPMEC (*p* < 0.01) and the 50 ± 11 observed in 600 k HPMEC (*p* < 0.05, Figure 2C). The total branching length of 100 k HPMEC at day 7 was 9866 ± 1244, which was about half the length in 300 k HPMEC (18,205 ± 2066 (*p* < 0.01)) and also half of the length in 600 k HPMEC (15,820 ± 1693 (*p* < 0.005)) (Figure 2D). Besides seeding density, culture time also affected VNF (Figure S1A). The number of nodes, number of master junctions, number of branches, and total branching length had all increased in all three cell densities after 7 days of culturing, and this difference remained at day 14.

#### 2.1.2. Vascular Structure in 3D Shown by the Live Cell Confocal Imaging System

Subsequently, 3D fluorescent imaging using 3D confocal laser scanning microscopy (Zeiss Cell Discover 7) was used to record the network formation of 600 k HPMEC on day 14 (Supplementary Video S1). The live cells were visualized by Calcein AM (green). Dead cells marked by PI were red, and all the nuclei of HPMEC marked by Hoechst were blue. The branches of the tubes stretched out in all three dimensions in the hydrogel. The video shows that live cells had stretched and formed tubes that connected with each other to form branches and meshes that expanded as a reticulated network through large parts of the hydrogel. Yet, the HPMEC-based networks were heterogeneously distributed throughout the hydrogel. The number of cells, as well as the number of tubes, increased gradually from the top to the bottom of the hydrogel. This implies that HPMEC had migrated towards the bottom during 3D culturing. At present, unfortunately, no reliable software exists to quantify vascular-like network features, such as tube length, from this type of video.

#### 2.1.3. Optical Coherence Tomography

**Figure 3:**
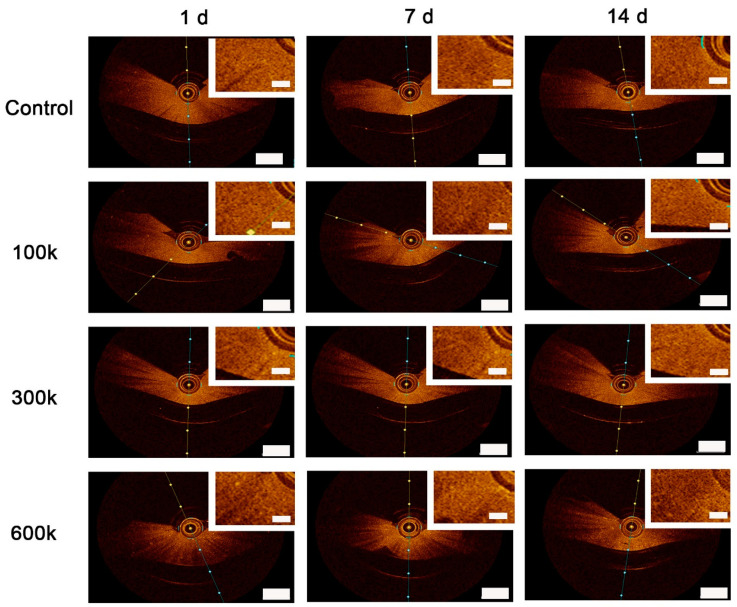
OCT analyses: HPMEC engaged in VNF in LV ECM hydrogels; 100 k, 300 k, and 600 k HPMEC seeded in LV ECM hydrogel examined after 1 day, 7 days, and 14 days. As control, hydrogels without HPMEC were used. Scale bars represent 1 mm (landscape view) and 0.2 mm (insets).

**Figure 4:**
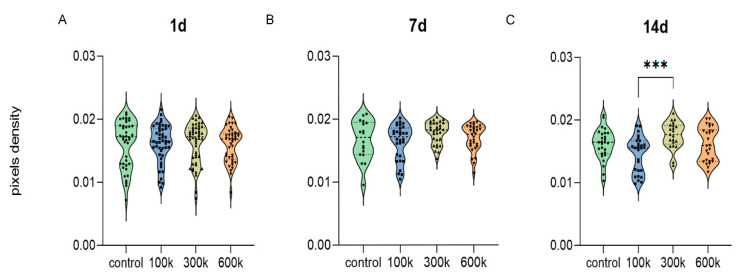
Densitrometric analyses of OCT imaging. HPMEC seeded in LV ECM hydrogels were compared at d1 (**A**), d7 (**B**), and d14 (**C**). Measurements were obtained from three pullbacks over each gel (*n* = 5, one way ANOVA comparing gel, *** *p* < 0.001).

In a clinical setting, optical coherence tomography is used to visualize the placement of stents in arteries. The difference in light diffraction between stents and arterial walls is sufficient to allow imaging by OCT. We set out to assess whether or not OCT allows for vascular networks and the structural protein networks of the hydrogels to be distinguished (Figure 3). The OCT-generated diffraction patterns were generated from three pullbacks of the imaging head on top of the gels. This generated depth images that showed speckled patterns. Bare and cell-loaded hydrogels appeared similar irrespective of culture time. By visual inspection of the OCT-generated output, vascular networks were indistinguishable from the diffraction caused by the hydrogels themselves. Quantification of the diffraction patterns through measurement of pixel densities also showed seeding density-dependence had no influence on measured diffraction. The only exception is that, at day 14, 300 k-seeded hydrogels (0.017 ± 0.0005) had a higher diffraction than 100 k-seeded hydrogels (0.015 ± 0.0006) (*p* < 0.001) (Figure 4C). Besides seeding density, culture time also affected average pixel density (Figure S1B). The average pixel density of 100 k HPMEC had decreased on day 14 (0.015 ± 0.0006) compared to day 1 (0.016 ± 0.0004) and day 7 (0.016 ± 0.0005) (*p* < 0.05). Hydrogels with 300 k seeded HPMEC showed an increased average pixel density at day 7 (0.018 ± 0.0003) compared to the day 1 (0.016 ± 0.0005), which remained unchanged at day 14. In hydrogels with the highest seeding density (600 k), no changes in diffraction were observed, nor were there changes in bare hydrogels over a period of 14 days (Figure S1B).

#### 2.1.4. Vascular-like Structure Analyses by H&E Staining

**Figure 5:**
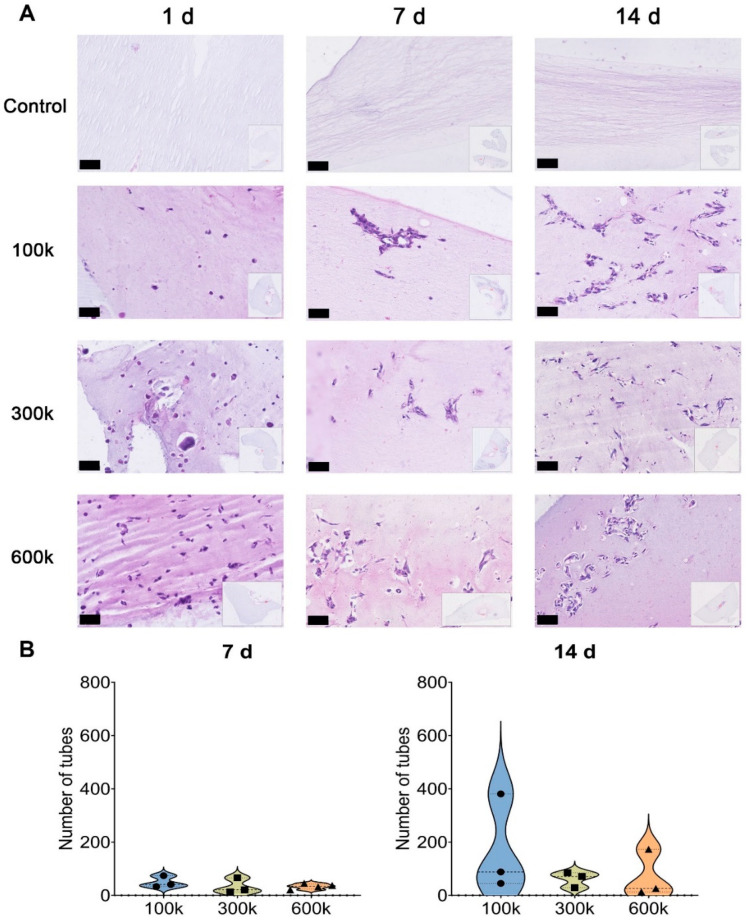
Histochemical analyses of VNF by HPMEC seeded in LV ECM hydrogels and stained by H&E. (**A**): 100 k, 300 k, and 600 k HPMEC seeded in LV ECM hydrogel were examined at days 1, 7, and 14 of culturing (original magnification was 40×) after H&E staining of thin sections. Bare hydrogels served as controls. Control hydrogels show the fiber-like meshwork of the LV ECM hydrogels. Cell-seeded hydrogels show clusters of cells (purple with dark-stained nuclei), often arranged as tubular structures. Insets show the entire section as scanned by the Hamamatsu image scanner. Scale bars represent 100 µm. (**B**): quantification of the number of tubes (visible as cell clusters with a lumen) at days 7 and 14 (*n* = 3, one way ANOVA comparing gel).

The distribution and arrangement of HPMEC in thin sections of ECM hydrogels were visualized by H&E staining. In the control cell-free hydrogels, fibers were discerned. These were organized as parallel bundles and homogeneously distributed through the hydrogels. A similar organization was observed between the cells in seeded hydrogels (Figure 5A). Consistent with inverted fluorescence microscopy imaging, HPMEC in a single section of the gel were rounded at one day after seeding (Figure 5A, left column), except that some HPMEC in the 600 k density were spindle-shaped. The seeded HPMEC had developed vascular-like networks by day 7, which appeared to have expanded by day 14 (Figure 5A, middle and right columns). It would seem that to engage in VNF, HPMEC had migrated through the hydrogel because, by eye, the cellular distribution at days 7 and 14 was more heterogeneous than one day after seeding. We quantified the number of ‘lumens’ per area because in a thin section it is obviously not possible to detect branching tubes in all directions. With this assay, the number of tubes at all three cell densities and at both day 7 and day 14 was similar, albeit that at day 14 more lumens tended to be present (Figure 5B,C). For 100 k HPMEC, the number of tubes was 49.3 ± 12.6 at day 7 versus 171.3 ± 105.6 at day 14. For 300 k HPMEC, the number of tubes was 33.3 ± 16.5 at day 7 versus 61.7 ± 16.8 at day 14. For 600 k HPMEC, there were 33.8 ± 5.0 tubes at day 7 and 70.7 ± 51.3 tubes at day 14. Additionally, the H&E-stained sections had a higher density of ECM near tubular structures, which was observed as a darker color.

#### 2.1.5. Collagen Is Deposited in the Close Vicinity of Vascular Networks

**Figure 6:**
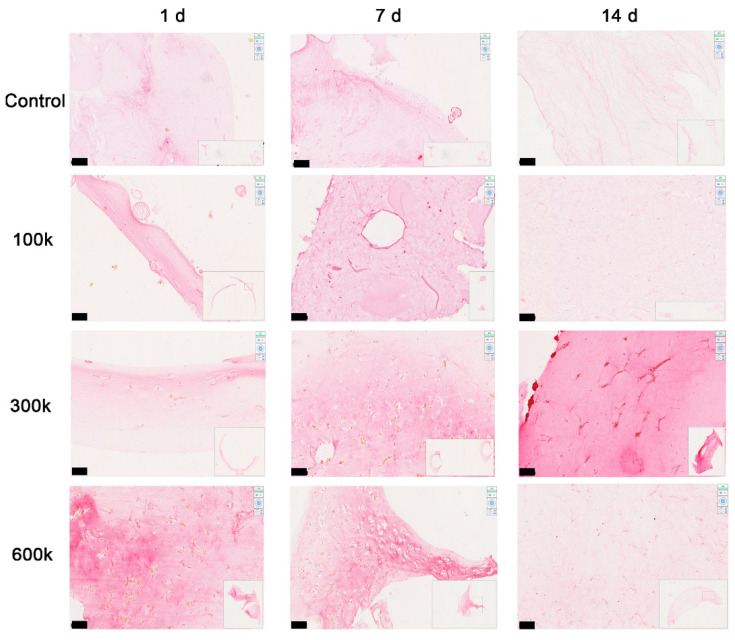
Influence on collagen organization of the VNF of HPMEC seeded in LV ECM hydrogels as examined by picrosirius red staining. Picrosirius red-stained sections of 100 k, 300 k, and 600 k HPMEC in LV ECM hydrogel at days 1, 7, and 14. As a control, bare LV ECM hydrogels were used. Staining intensity was markedly increased in the vicinity of cell clusters and tube-like structures in a seeding density-dependent fashion, while more distal of cells staining was weaker. The original magnification used for scan slides (insets) in Hamamatsu imaging was 20×. Scale bars represent 100 µm.

**Figure 7:**
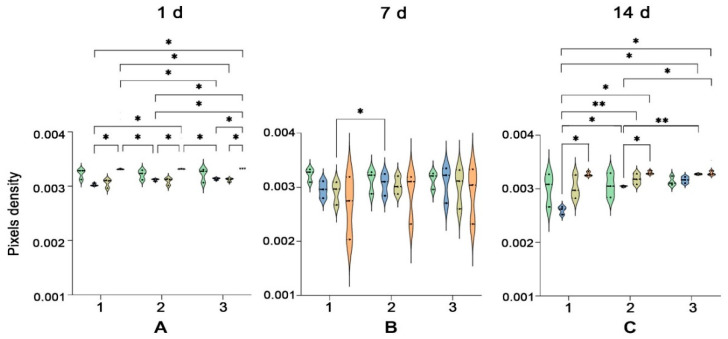
Quantitative analyses of picrosirius red staining imaging. Densitrometric data acquired using ImageJ for days 1 (**A**), 7 (**B**), and 14 (**C**) were compared with regard to seeding density (100 k, 300 k, 600 k, and control (no cells)). Three ROI are depicted: 1—close to cells, 2—at an intermediate distance from cells, and 3—cell-free area. Data represent *n* = 3 independent experiments assessed with two-way ANOVA multiple comparisons tests. * *p* < 0.05, ** *p* < 0.01.

To better investigate the changes in ECM density during VNF, picrosirius red staining was performed to reveal collagen fibers. New collagen was mostly deposited in the close vicinity of vascular networks after day 7 (Figure 6), and also on day 14. More collagen was deposited after networks formed compared to the collagen in the gel on day 1, though collagen distribution was heterogeneous. This heterogeneity was also observed in control gels and likely caused by incompletely degraded ECM fragments. The pixel density of 100 k HPMEC (0.0030 ± 1.35 × 10^−5^) is less than 600 k HPMEC (0.0033 ± 4.33 × 10^−6^) close to cells (ROI 1) on day 1 (Figure 7A). Additionally, the pixel density of 100 k HPMEC (0.0026 ± 4.29 × 10^−5^) was less than 600 k HPMEC (0.0033 ± 2.34 × 10^−5^) near cells on day 14 (*p* < 0.05) (Figure 7C). Further, more distal from cells but not in areas totally devoid of cells (ROI 2), the pixel density of 100 k HPMEC (0.0030 ± 7.31 × 10^−6^) was lower than 600 k HPMEC (0.0033 ± 1.90 × 10^−5^) on day 14 (*p* < 0.05) (Figure 7C). The collagen density in controls and in cell-free areas (ROI 3) did not change over time, irrespective of cell seeding density. In general, more collagen was deposited in a culture time- and seeding density-dependent fashion.

**Figure 8:**
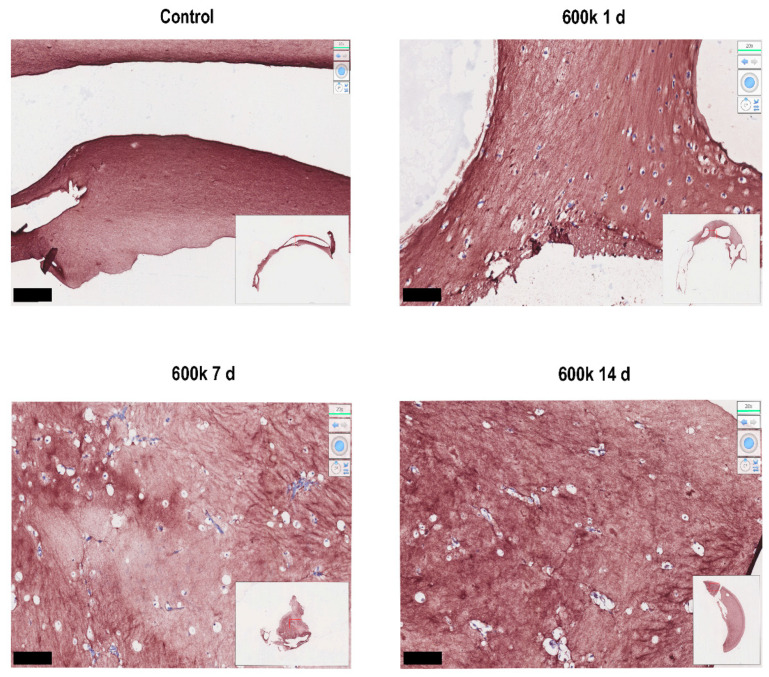
Immunohistochemical staining for the presence of collagen type I. LV ECM hydrogels were seeded with 600 k HPMEC and assessed at days 1, 7, and 14 for collagen type I. Bare hydrogels served as controls. Micrographs shown are areas with representative VNF taken from scans by Hamamatsu imaging at 20× magnification (insets). Collagen fibers are visible as a fibrous meshwork, while cells are white with a blue nucleus.

As expected, immunostaining for collagen type I showed its presence throughout the gel (Figure 8) because the original LV ECM hydrogel (Figure 8, control) was rich in collagen [6]. Irrespective of cell presence or culture time, a distinct fibrous collagen network was present. At days 7 and 14, these collagen networks were more dense and particularly present in VNF areas, suggesting they were newly deposited (Figure 8).

### 2.2. Discussion

The purpose of this study was to compare different analysis modalities to assess vascular-like network formation by endothelial cells in cardiac-derived LV ECM hydrogels. We assessed four distinct methods to visualize angiogenesis and the matrix remodeling in cardiac ECM hydrogels at three cell densities at three time points. Our results showed that cell density and culture time affect VNF, and different techniques, having their own advantages and limitations, provide a complementary view of vascularization in cardiac hydrogels.

#### 2.2.1. Technical Clues

We previously showed that human umbilical vein endothelial cells (HUVEC) cocultured with ASC show 3D VNF in LV ECM hydrogels [6]. At that time, imaging of these 3D structures was poor at best, yet sufficed to yield a proof-of-concept. To better visualize VNF in LV ECM, we used inverted light/fluorescence microscopy. The quantification of branches and nodes in VNF was analyzed by densitometric analysis software, i.e., ImageJ. Inverted microscopy not only visualized VNF by HPMEC in LV ECM hydrogels, but also gave a hint that HPMEC had distributed in a heterogeneous fashion during culture as a consequence of VNF. However, the relatively high opacity of ECM hydrogels and the shallow DOF of the inverted microscope limited the observation, though production of reproducible micrographs for analyses was possible. Since the VNF was in all directions through the ECM hydrogel, using one focus point to visualize VNF hampered visualization in lower layers and other directions. Focus stacking would be a solution to generate a higher DOF, but with this type of ‘simple’ benchtop microscope that is impossible. Moreover, stacking of images from a 3D environment into a ‘flat’ 2D image means that, for networks, one cannot distinguish between genuine branch points and tubes that cross at different gel depths.

The use of a confocal laser scanning microscope in combination with a single wavelength, such as in the live cell confocal imaging system which is dedicated to scanning along the Z axis, enabled the reconstitution of 3D reconstructions of VNF. It solved the deficiencies of an inverted microscope (EVOS). Since the Cell Discover system supports cell culture during imaging, the long scanning duration does not (and did not) negatively affect cell survival. Multi-focus point imaging allowed relatively fast and detailed imaging of VNF in the whole gel at the cost of reducing step size to approx. 300 µm. Higher resolution imaging is possible, though at the cost of decreasing the volume that can be scanned, which compromises the imaging of larger branching structures. It would also require long scanning times (hours per image), such that the scanning of multiple samples under different experimental conditions would be challenging if not impossible. Our fast scanned images had acceptable resolution in relation to tubes and cells and were an improvement over inverted fluorescence microscopy (EVOS)-generated micrographs, albeit less user-friendly or amenable to quantitative analyses. At present, adequate, accurate, and reliable algorithms to quantify the live cell confocal imaging system-generated reconstructions (such as 3D networks) have not been developed for the confocal system due to a lack of 3D reconstitution and analysis software. For EVOS image quantification, this proved less of a problem because the 3D images were projected in a 2D image suitable for analysis with ImageJ. These 3D reconstructions using the live cell confocal imaging system do not allow for such a 2D projection, which means that comparing branches, master junctions, and nodes between groups cannot be achieved.

Refraction patterns generated by the noninvasive technique of OCT during scanning of cast ECM hydrogels would, in principle, allow for visualization of changes in matrix density, such as changes caused by the presence of cells or changes in architecture. Unlike the inverted fluorescent microscope, the light from probe emissions penetrates hundreds of micrometers. Therefore, with OCT, we could scan the entire gel. Pixel density reflected not only the variations in VNF among the three groups, but also the difference in the ECM caused by cell behavior (collagen secretion, ECM remodulation, etc.). In our hands, the resolution of OCT was too low to differentiate between single cells, vascular-like structures, and ECM (changes) at all time points. We used OCT equipment that is clinically used to visualize and inspect the pathologies of airways in COPD patients. This included changes in the airway matrix; therefore, the fact that we assessed a low matrix density ECM (2% *w*/*v* only), and that cells poorly refract light, likely caused the inability to assess processes with high enough sensitivity. This corroborates data from our group that µMRI and µCT have too low of a resolution to distinguish between the ECM and cells (unpublished results).

While the real-time imaging methods mostly provided information about the ‘whereabouts’ of cells in time, the immunochemical-stained sections provided quantifiable information on ‘what’ had happened. The presence and arrangement of ECM fibrils was readily visible after both H&E and picrosirius red staining. Cells and tubes were most visible after H&E staining, but matrix changes were more visible after picrosirius red staining. In fact, accumulation of new matrix and/or remodeling of cast hydrogel matrix was readily seen as changes in color density that could be densitrometrically quantified as pixel density. This showed that, during culture, matrix was remodeled by endothelial cells in a seeding density-dependent and time-dependent fashion. Live fluorescence microscopy showed that, over time, vascular-like networks developed in a seeding density-dependent fashion. Indeed, this was corroborated by H&E staining, which showed an increased number of tubes over time, irrespective of seeding density. The stained sections also showed that a majority of the tubes lay parallel to the cutting direction. This suggests that the fibers of the ECM were (re)arranged in a parallel fashion, and that the vascular tubes followed them. Indeed, immunohistochemical staining for collagen type I showed the more or less parallel alignment of fibers in large parts of the hydrogels. This emphasizes the relevance and feasibility of using distinct complementary imaging methods to visualize, qualify, and quantify vascular-like networks in ECM-derived hydrogels.

#### 2.2.2. Cell Behavior in 3D Clues

The complementary application of the techniques revealed that VNF by HPMEC was time- and seeding density-dependent. Higher densities were prone to forming networks as early as day 7. The lowest seeding density engaged in VNF later, while the networks remained stable for longer. A limitation of our system might be that HPMEC are immortalized cells, and their urge to proliferate surpasses the formation of stable vascular networks. In fact, the vascular-like networks often disintegrate upon prolonged culturing due to extensive proliferative overgrowth (data not shown). The advantage of HPMEC is that vascular-like networks are established without the need for pericytic cells, as we showed to be essential for HUVEC [7]. As stated above, the observed vascularization was accompanied by the extensive remodeling of ECM. This corroborates our earlier observations that mesenchymal cells embedded in collagen-mimicking hydrogels respond to the matrix by remodeling it [21,22].

The use of complementary techniques also revealed that, in cardiac LV ECM hydrogels, matrix remodeling occurred in the vicinity of the tubes and consisted of the deposition of condensation from collagens. Whether ECM was also degraded was not a topic of our research but appears feasible, as endothelial tip cells secrete MMP9. The molecular mechanism is part of current studies.

Interestingly, our study opens new ways of treating hypoxically damaged tissues, (after myocardial infarction, for example). Cardiac hydrogels preceded with endothelial cells or vascular-like networks administered intramyocardially might facilitate formation of microvascular networks and (fast) reperfusion to augment healing.

## 3. Conclusions

Based on our results, the benefits and limitations of the individual methods of assessing vascular-like network formation in ECM hydrogels are listed in Table 1.

## 4. Materials and Methods

### 4.1. Hydrogel Synthesis

Porcine hearts were purchased from a slaughterhouse (Kroon Vlees, Groningen, The Netherlands). Pigs were approx. 14 to 16 weeks old. Left ventricles were excised and dissected into 1 cm^3^ pieces. The tissue was mixed with ice-cold Dulbecco′s phosphate-buffered saline (DPBS) (Lonza Walkersville, Inc., Walkersville, MD, USA), and then minced in a kitchen blender (Bourgini, Breda, The Netherlands) with DPBS to further fragment the tissue. The tissue homogenate was sonicated at 100% power for 1 min. The tissue was collected by centrifugation, washed twice, and incubated with 0.5% trypsin (Thermo Fisher Scientific, Waltham, MA, USA) at 37 °C under constant shaking for 4 h. After twice washing with PBS, the slurry was incubated in Milli-Q^®^ water under constant shaking overnight.

Next, the tissue homogenate was treated with excess saturated NaCl (6M) for 3 h, followed by repeated washing in PBS. Subsequently, the homogenate was incubated in 1% SDS (Sigma-Aldrich, St. Louis, MO, USA) and 1% Triton X-100 (Sigma-Aldrich), followed by 1% sodium deoxycholate (Sigma-Aldrich) with extensive and repetitive washing between incubations. To remove residual DNA, the homogenate was incubated with 30 µg/mL DNase (Roche Diagnostics GmbH, Mannhelm, Germany) in 1.3 mM MgSO_4_ and 2 mM CaCl_2_. All incubations were under shaking at 37 °C overnight; the homogenate was washed with MilliQ^®^ water at intervals between each treatment for 10 min three times. Finally, the homogenate was washed with DPBS six times, kept under constant shaking for an hour each time. Last, the homogenate was collected after centrifugation, sterilized with 70% ethanol overnight, and washed and stored in sterile MilliQ water at −20 °C. The LV ECM samples were snap-frozen in liquid nitrogen and lyophilized with a FreeZone Plus lyophilizer (Labconco, Kansas City, MO, USA) and ground to a fine powder with an ULTRA-TURRAX (IKA, Staufen, Germany). A total of 20 mg/mL of ECM powder was then digested with 2 mg/mL of porcine pepsin (Sigma-Aldrich, St. Louis, MO, USA) in 0.01M of HCl under constant agitation at room temperature for 6 h. After digestion, the digested ECM was neutralized by addition of 1/10th volume 0.1M NaOH and subsequently 1/10th volume 10xDPBS to generate an isotonic and neutral pH ECM pregel.

### 4.2. 3D Cell Culture

Specified numbers of HPMEC (100,000 (100 k), 300,000 (300 k), and 600,000 (600 k)) were collected by centrifugation and resuspended in approx. 50 µL of RPMI-1640 (Lonza, Basel, Switzerland). The resuspended cells were thoroughly, yet gently, mixed with 200 µL of cardiac LV ECM pregel while avoiding trapping of air bubbles and ensuring homogeneous mixing of the HPMEC with the pregel. The cell–gel mixtures were cast into individual wells of 48-well plates and incubated in the 37 °C incubator for 40 min, after which the gels had solidified. Next, 500 µL of endothelial culture medium was added, comprising RPMI-1640 with 20% heat-inactivated fetal bovine serum (FBS, Sigma-Aldrich, MO, United States), 1% penicillin/streptomycin (#15140122, Gibco Invitrogen, Carlsbad, CA, USA), 1% L-glutamine (#17-605E, Lonza Biowhittaker, Verviers, Belgium), 5 U/mL heparin (LEO Laboratories Limited, Ballerup, Denmark), and 30 µg/mL of endothelial growth factors (home-made bovine brain extract).

### 4.3. Fluorescent Microscopy (2D)

**Figure 9:**
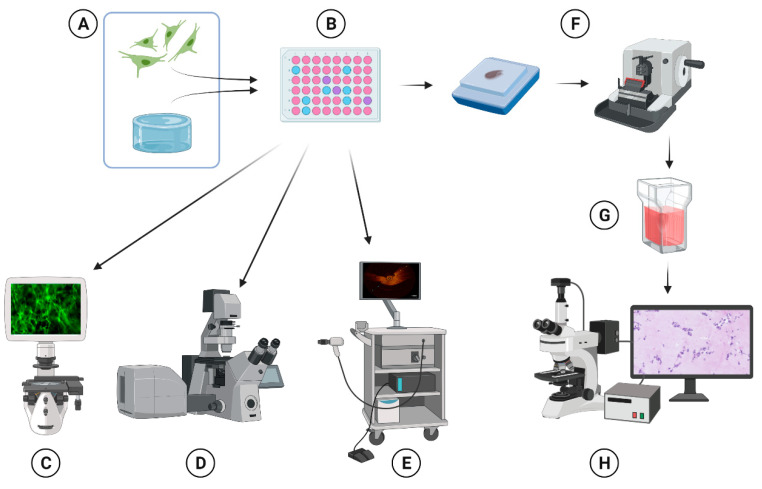
Schematic representation of the methodology. HPMEC were mixed with pregel (**A**) and cast in 48-well plates at 37 °C (**B**). Visualization of cells was performed via inverted fluorescence microscopy (**C**), the use of a live cell confocal imaging system (**D**), and OCT (**E**). After culture, the gels were fixed with paraformaldehyde, embedded into the paraffin, and thin-sectioned (**F**). The sections were subjected to different (immuno)histochemical staining (**G**) and imaged by microscopy (**H**). [Image partially made with BioRender.] [23].

After 1, 7, and 14 days of culture, the cells were incubated at 37 °C in the dark with Hoechst nuclear dye (1:50, Thermo Fisher Scientific, Waltham, MA, USA), Propidium Iodide (PI, 1:500, Thermo Fisher Scientific, Waltham, MA, USA), and Calcein AM (1:200, Thermo Fisher Scientific, Waltham, MA, USA) vital stain for 30 min. The wells were washed with PBS twice and imaged with an inverted fluorescent microscope (EVOS model M5000, Thermo Fisher) (Figure 1) using the following filters: DAPI, GFP, and Texas Red. At least three random areas were selected to make fluoromicrographs in all three channels (Figure 9).

Vascular-like network formation (VNF) analyses: We adopted the ImageJ built-in function to subtract the background of pictures with EVOS: process, subtract, background, and separate colors. EVOS images were analyzed with the Endothelial Tube Formation Assay—angiogenesis analyzer in ImageJ.

### 4.4. Live Cell Confocal Imaging System

On day 14, cultures of 600 k HPMEC in ECM hydrogel were stained with Hoechst, PI, and Calcein AM as described above, and optical sections were obtained by scans using the Zeiss Cell discoverer 7 imaging system (Zeiss, Jena, Germany). Optical settings were an objective lens magnification of 5× and an Optovar magnification of 1×. The interval of the scanning was 325 µm. Detection wavelengths (excitation–emission) were 400–565 nm for Hoechst, 400–575 nm for Calcein AM, and 575–700 nm for Texas Red. Image stacks and videos were generated using Zeiss Zen 3.3 software.

### 4.5. Optical Coherence Tomography

Gels were carefully removed from the wells, placed on top of a plastic Petri dish, and scanned with a real-time optical coherence tomography (OCT) device, Ilumien™ Optis (St. Jude Medical, Zaventem, Belgium), using a Dragonfly™ Duo Imaging catheter (St. Jude Medical, Zaventem, Belgium). The Ilumien™ Optis generates infrared-based cross-sectional images [24]. The imaging catheter was placed on top of the gel and two pullbacks (scans) were performed at 90° and 180° orientation. Three images were randomly chosen for analysis from the video taken by OCT in one pullback. Regions of interest (ROI) were all selected in the area close to the center of the imaging catheter. The pixel density of ROI for all OCT images was analyzed using ImageJ Ver 1.52p [25] through basic intensity quantification with ImageJ.

### 4.6. Immunohistochemical Analyses

For hematoxylin and eosin (H&E) staining, 4 µm thin paraffin sections were deparaffinized and stained with hematoxylin for 10 min. Slides were rinsed with tap water till the water was clear. Slides were then transferred to a Coplin jar with eosin solution for 3 min and washed with 70% ethanol for 20 s, 90% ethanol for 20 s, 100% ethanol for 1 min, and xylene for 3 min.

For picrosirius red staining, thin sections were deparaffinized, waxed, and hydrated. First, nuclei were stained with hematoxylin for 8 min. After being washed for 10 min in running tap water, slides were incubated in picrosirius red solution for one hour, followed by two washes in acidified water. Slides were dehydrated with three changes of 100% ethanol, and were then cleared in xylene and mounted in a resinous medium. For collagen type I immunochemical staining, deparaffinized and hydrated thin sections were heated in 10 mM Tris/ HCl buffer at 85 °C overnight for antigen retrieval. Next, sections were incubated with 0.3% hydrogen peroxide (Merck, Darmstadt, Germany) in PBS at room temperature for 30 min. Slides were incubated with the mouse-anti-human col1a1 (abcam88147) primary monoclonal antibody, diluted to a ratio of 1:300 in PBS/1% BSA for 1 h. After PBS washes, the sections were incubated with peroxidase labeled rabbit-anti-mouse (Thermo Fisher Scientific, catalog #PA1-28568) for 30 min. To increase detection sensitivity, a peroxidase labeled goat-anti-rabbit (Thermo Fisher Scientific, catalog #A32731) was applied on the slides for another 30 min. Color development was achieved using the NovaRed staining kit for 8 min.

The picrosirius red staining micrographs were analyzed by FIJI through basic intensity quantification in ImageJ, the same method as used for OCT analysis. Three ROI of the same size were marked as Region 1, 2, and 3. Region 1 represented areas with a high density of HPMEC. Region 2 represented areas of medium HPMEC density. While region 3 represented areas devoid of cells. The averaged pixel density per area was determined for all ROI.

### 4.7. Statistical Analysis

All statistical analyses were performed using GraphPad Prism v9.2.0 (GraphPad Company, San Diego, CA, USA). All data were scrutinized for outliers using the robust regression and outlier removal (ROUT) test. Fluorescent microscopy imaging data and picrosirius red staining micrograph data were analyzed with two-way ANOVA. OCT imaging data and histochemistry data were analyzed with one-way ANOVA and corrected multiple comparisons with the Tukey post-hoc test. Differences were considered significant at *p* < 0.05 in the corresponding statistical tests.

## Figures and Tables

**Table 1:** Comparison of benefits and limitations of four methods of assessing VNF in ECM hydrogels.

*Method*	Benefit	Limitation
*Inverted fluorescence microscopy*	Real-time method Detect changes in cell density over time Can visualize VNF Quantifiable micrographs	Low DOF Micrographs are surrogate 3D
*Confocal microscopy*	Real-time method VNF monitoring over time 3D micrographs and scanning videographs High resolution up to hundreds of µm	No software to quantify 3D structures Time consuming to scan at high resolution
*OCT*	Real-time method 3D imaging	Low resolution VNF not visible
*(Immuno)histochemistry*	High resolution Quantifiable Detection of cells and VNF Imaging of matrix organization (collagens)	Destructive method Tedious 3D reconstruction

## Data Availability

Not applicable.

## Supplementary Materials

The following supporting information can be downloaded at: https://www.mdpi.com/article/10.3390/gels8110729/s1, Figure S1: Influence of culture time on VNF by HPMEC in LV ECM hydrogels. A: Analysis of fluorescent microscopy imaging. B: Analysis of OCT imaging; Video S1: 600 k HPEMC on day 14 scanned by cell discoverer 7.
