# Supplementary material for: From Macro to Micro: Comparison of Imaging Techniques to Detect Vascular Network Formation in Left Ventricle Decellularized Extracellular Matrix Hydrogels
Source: Gels. 2022 Nov 10;8(11):729. doi: 10.3390/gels8110729 (PMC9689814; doi:10.3390/gels8110729)
Supplement: Supplementary file 1 [file gels-08-00729-s001.zip › Supplementary Data.pdf]

Supplementary Data

A

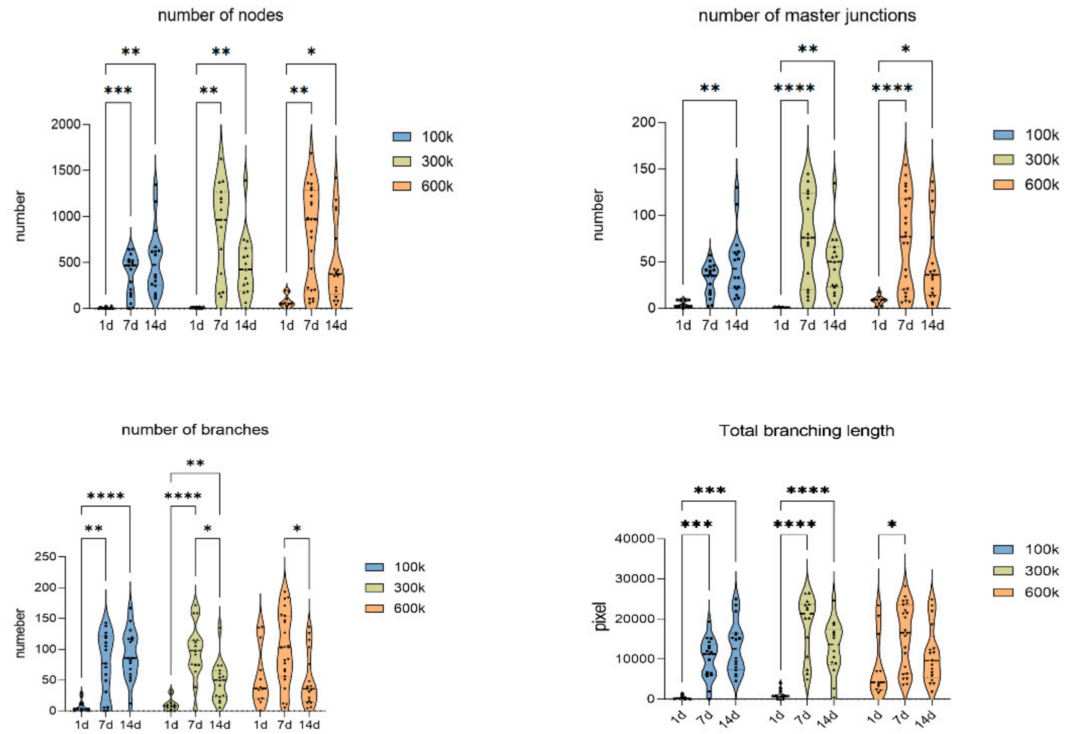

B

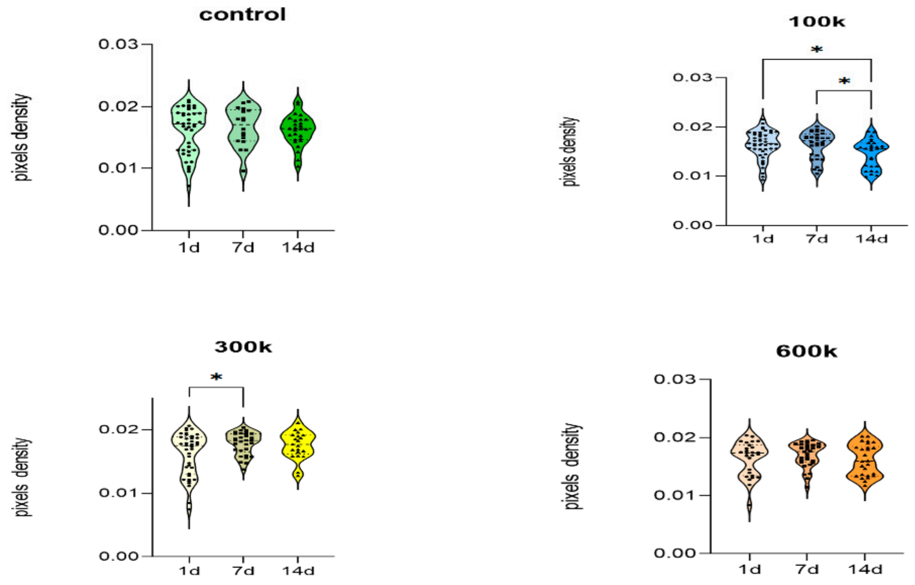

Figure s1

Fig. S1. *Influence of culture time on VNF by HPMEC in LV ECM hydrogels*
*A: Analysis of fluorescent microscopy imaging. Comparison of number of nods, number of master junction, number of the branches and total branching length based on ImageJ quantification among 3 time point 1d, 7d and 14d of 3 cell density 100k, 300k and 600k. (n=5, each sample was taken photos on 3 ROIs. One way ANOVA comparing gel, *p<0.05, **p<0.01, ***p<0.001.).*

*B: Analysis of OCT imaging. Statistic analyses of pixel density based on ImageJ quantification. Measurements were obtained from 3 pull backs over each gel(n=5, one way ANOVA, *p<0.05,***p<0.001).*
